# Impact of Sex and Smoking on the Efficacy of EGFR-TKIs in Terms of Overall Survival in Non-small-Cell Lung Cancer: A Meta-Analysis

**DOI:** 10.3389/fonc.2020.01531

**Published:** 2020-08-25

**Authors:** Jian Xiao, Liang Zhou, Bixiu He, Qiong Chen

**Affiliations:** ^1^Department of Geriatrics, Respiratory Medicine, Xiangya Hospital of Central South University, Changsha, China; ^2^National Clinical Research Center for Geriatric Disorders, Xiangya Hospital of Central South University, Changsha, China; ^3^Department of Critical Care Medicine, People's Hospital of Ningxia Hui Autonomous Region, Yinchuan, China; ^4^Department of Geriatrics, People's Hospital of Ningxia Hui Autonomous Region, Yinchuan, China; ^5^Ningxia Geriatrics Center, Yinchuan, China

**Keywords:** meta-analysis, epidermal growth factor receptor-tyrosine kinase inhibitor, overall survival, non-small-cell lung cancer, sex

## Abstract

**Background:** To comprehensively understand the impact of sex and smoking on the efficacy of epidermal growth factor receptor-tyrosine kinase inhibitor (EGFR-TKI) therapy in terms of overall survival (OS) in non-small-cell lung cancer (NSCLC).

**Methods:** PubMed, Cochrane Library, Embase, and Scopus were searched from inception to March 17, 2019. OS was analyzed based on hazard ratios (HRs) and 95% confidence intervals (CIs) and estimated using the random effects model.

**Results:** Our meta-analysis included 22 studies involving 11,874 patients. In the primary analysis, we found no statistically significant efficacy difference for EGFR-TKI intervention between females and males (pooled HR 0.95, 95% CI 0.87–1.04, *P* = 0.30) and no obvious efficacy difference between never smokers and ever smokers (pooled HR 0.91, 95% CI 0.76–1.09, *P* = 0.31). In the subgroup analysis of placebo control treatment, we found that female NSCLC patients who received EGFR-TKI therapy had a longer OS than male patients (pooled HR 0.86, 95% CI 0.75–1.00, *P* = 0.04), while smoking status showed no significant effect on the efficacy of EGFR-TKI treatment in terms of the OS of NSCLC patients in all subgroup analyses.

**Conclusion:** The efficacy of EGFR-TKI therapy for NSCLC patients is independent of smoking status but dependent on sex, and females have a longer OS than males.

## Introduction

Lung cancer is one of the most common cancers in men and women, and there is no doubt that lung cancer poses the greatest threat to human life, as it results in one-quarter of all cancer deaths (1). However, non-small-cell lung cancer (NSCLC) accounts for more than 85% of lung cancer, (2) and it is well-known that there is a significant difference in the development of NSCLC between male and female patients and between patients of different smoking statuses (3, 4).

Overactivation of epidermal growth factor receptor (EGFR) tyrosine kinases is a key mechanism leading to the development of NSCLC (5). In recent years, epidermal growth factor receptor-tyrosine kinase inhibitors (EGFR-TKIs) have achieved good clinical efficacy in the treatment of NSCLC. At present, three generations of EGFR-TKIs have been widely used in the clinical treatment of NSCLC, such as gefitinib, afatinib, and osimertinib, which represent first-, second-, and third-generation EGFR-TKIs, respectively (6). Of course, other new EGFR-TKIs are also on the way to development and being promoted (7). There is no doubt that EGFR-TKI therapy plays a pivotal and irreplaceable role in the treatment of patients with NSCLC.

Previous meta-analyses focused on EGFR-mutated NSCLC patients and the progression-free survival (PFS) have concluded that female and non-smoking NSCLC patients have better efficacy with EGFR-TKIs than male patients and smokers (8–10). However, as we know that EGFR-TKIs treatment also shows some kind of treatment effects for NSCLC patients with unknown or wild-type EGFR status, and the association of overall survival (OS) with EGFR-TKIs treatment in NSCLC patients. Those previous studies could be expanded.

In the meantime, we found two meta-analyses that comprehensively and thoroughly studied the effect of sex on the efficacy in terms of OS of immune checkpoint inhibitors in cancer treatment (11, 12). We were very interested in these two studies, which prompted us to re-examine the impact of sex and smoking status, the two most common and important clinical features in NSCLC, on the efficacy of EGFR-TKIs. Thus, we have done and now report a meta-analysis of the association of sex and smoking with the efficacy of EGFR-TKIs in terms of OS in NSCLC.

## Methods

### Search Strategy and Selection Criteria

We performed this meta-analysis according to the PRISMA guidelines (13). PubMed, Cochrane Library, Embase, and Scopus were searched from inception to March 17, 2019. Two authors independently searched the databases. The main search terms were “lung cancer,” “survival,” “hazard ratio,” “EGFR” and “randomized controlled trials.” Full details of our search strategies for the databases are shown in the Supplementary Material (Supplementary Content 1). Titles, abstracts and full-text articles were reviewed independently by two authors. Inconsistencies were discussed by all authors to reach consensus. Reference lists were also reviewed to identify additional relevant studies.

The literature inclusion criteria were as follows: randomized controlled clinical trials for NSCLC that contained any single EGFR-TKI treatment; the treatment plans in the corresponding control group did not contain any other EGFR-TKIs; the prognosis endpoint was OS, and the corresponding hazard ratios (HRs) and 95% confidence intervals (CIs) were reported according to sex and/or smoking status; and the full-text manuscripts were published in English. The exclusion criteria were as follows: retrospective studies of clinical cases; abstracts, reports, and papers from conferences; literature reviews and meta-analyses.

### Data Extraction and Study Quality Assessment

Two authors independently extracted data from the included studies. Discrepancies were resolved by all authors through discussion to reach consensus. The following variables were extracted from each study: first author, publication year, EGFR mutation status, trial name, lines of therapy, EGFR-TKI intervention drug, control treatment plan, total number of patients, median age (years), median follow-up time (months), overall HR with 95% CI, HR with 95% CI according to patient sex, and HR with 95% CI according to smoking status. When duplicate publications were identified from one trial, we included only the most complete report.

The methodological quality of the included studies was assessed using the five-point Jadad score (14), which judges manuscripts according to the descriptions of randomization, blinding and withdrawals and dropouts. The details are as follows: whether randomized or not; whether randomization was described or not; whether double-blinded or not; whether blinding was described or not; and whether withdrawals and dropouts were described or not. For each of the above questions, if the answer is yes, the study gets 1 point; if the answer is no, the study gets 0 points. The quality scale ranges from 0 to 5 points for each controlled trial. A score of 2 or less indicates a low-quality study, while a score of 3–5 indicates a high-quality study.

### Data Analysis

The HRs and 95% CIs were extracted from each study according to the classification of overall HR, HR in male patients, HR in female patients, HR in never smokers and HR in ever (former and/or current) smokers. For the overall HRs, we used the random effects model to calculate the pooled HR directly. For the HRs classified by sex and smoking status, we first calculated the interaction HRs and 95% CIs for each study and thereafter obtained the pooled HR using the random effects model. The heterogeneity between studies was identified using the Q-test and quantified using *I*^2^-values (11, 12). Potential publication bias was evaluated using the Egger and Begg test. To assess the differences between males and females or never smokers and ever smokers, we performed calculations using log HR to evaluate whether the variations differed from the null hypothesis by using the χ^2^-test (11, 12).

We performed subgroup analyses to further explore the variation of the effect of sex and smoking status on EGFR-TKI therapy efficacy. We only considered subgroups that included no less than two studies. The subgroups were EGFR status (unknown, wild-type, and mutation), lines of therapy (>1 and 1), EGFR-TKI intervention (gefitinib, erlotinib, and others), and control treatment (placebo, chemotherapy and others).

We performed all data analyses using Stata 14.0 (StataCorp LP, USA). All reported *P-*values are 2-sided, and a *P-*value of 0.05 indicated statistical significance. An HR < 1 indicated that EGFR-TKI efficacy was better than non-EGFR-TKI efficacy, EGFR-TKI efficacy in females was better than in males, and EGFR-TKI efficacy in never smokers was better than in ever smokers.

## Results

### Literature Search

**Figure 1:**
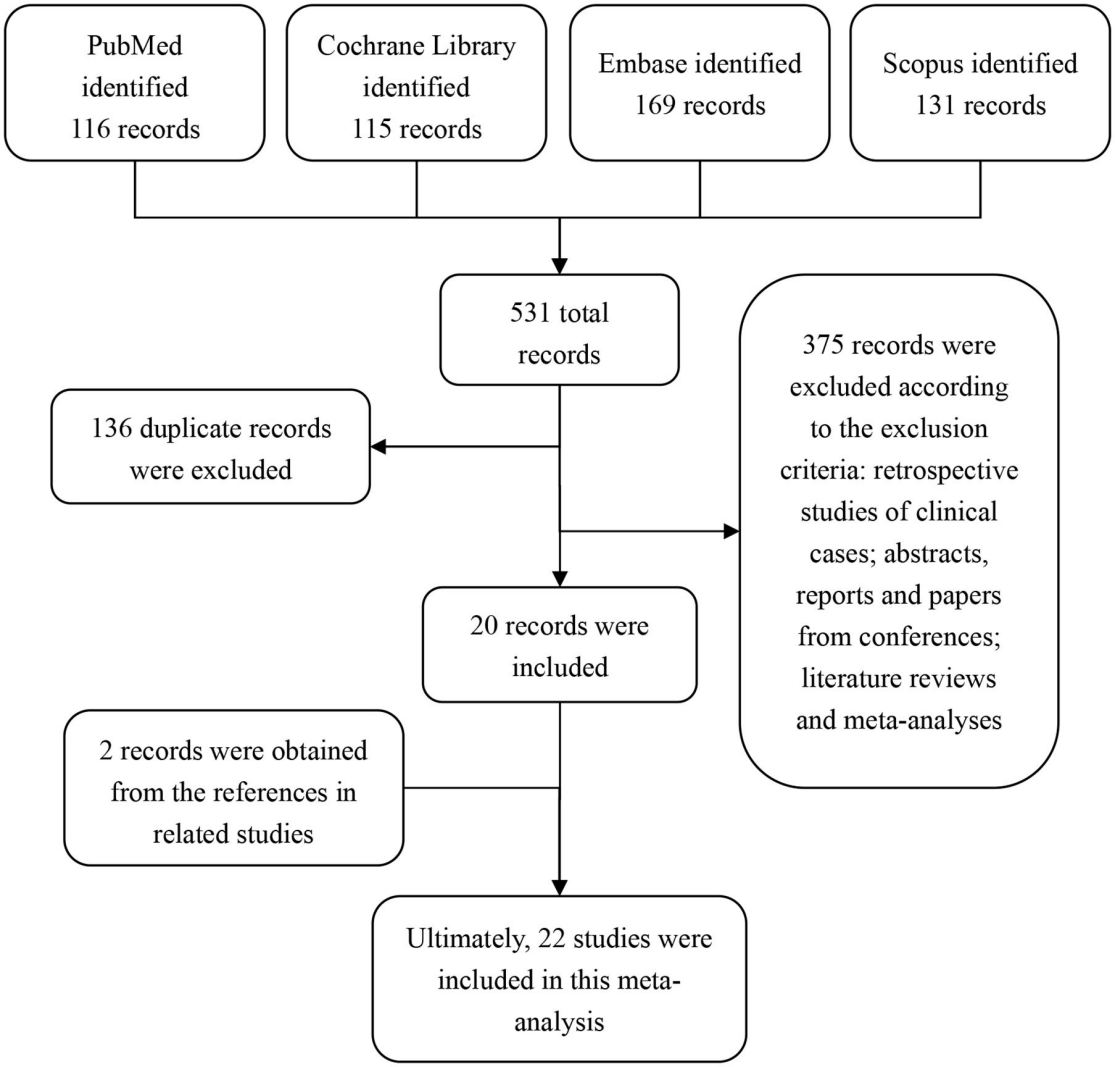
Flow diagram of the study selection.

By searching our search terms in the database, we obtained 531 potential publications. A total of 136 records were excluded because of duplicated titles. By reviewing the abstract and full text, 375 records were further excluded according to our inclusion and exclusion criteria. Therefore, 20 publications were selected. In addition, by reviewing the references from these 20 selected studies, we found an additional 2 studies that were also in line with our inclusion criteria. Ultimately, 22 studies (15–36) were included in this meta-analysis (Figure 1).

### Characteristics of the Identified Studies

We assessed the quality of the 22 studies by the Jadad score. The mean score was 3.64 (ranging from 3 to 5), and no study received a low-quality score (scored 2 or less), indicating that these included studies possessed high methodological quality. The Jadad scores for each study are listed in the Supplementary Table S1 in the Supplementary Material).

As shown in Table 1, for the 22 included studies, 3 involved patients with wild-type EGFR, and 5 involved patients with EGFR mutations, and the other 14 studies did not consider EGFR mutations in patients, which we defined as EGFR unknown. Eight of 22 studies were for first-line treatment, and 14 of 22 studies were for second-line or beyond treatments. Compared with placebo or standard chemotherapy, the EGFR-TKI interventions included gefitinib, erlotinib, dacomitinib, afatinib, and icotinib. In total, 11,874 patients were involved in these included trials.

**Table 1:** Main characteristics and results of the 22 studies included in the meta-analysis.

**Article source**	**EGFR situation**	**Trial name**	**Lines of therapy**	**EGFR-TKI intervention**	**Control treatment**	**Total patients**	**Median age (years)**	**Median follow-up (months)**	**Overall HR (95% CI)**	**Sex of HR (**95% CI**)**		**Smoking of HR (95% CI)**	
										**Male**	**Female**	**Never**	**Ever**
Thatcher et al. (15)	Unknown	ISEL	>1	Gefitinib	Placebo	1,692	62/61	7.2	0.89 (0.77–1.02)	0.91 (0.78-1.06)	0.77 (0.60-0.97)	0.67 (0.49-0.91)	0.92 (0.80-1.05)
Tsao et al. (16)	Unknown	BR.21	>1	Erlotinib	Placebo	731	62/59	NR	0.70 (0.58–0.85)	0.76 (0.62–0.94)	0.80 (0.59–1.07)	0.42 (0.28–0.64)	0.87 (0.71–1.05)
Kim et al. (17)	Unknown	INTEREST	>1	Gefitinib	Docetaxel	1,433	61/60	7.6	1.02 (0.91–1.15)	1.08 (0.95–1.24)	0.95 (0.78–1.17)	0.93 (0.70–1.23)	1.05 (0.92–1.19)
Maruyama et al. (18)	Unknown	V-15-32	>1	Gefitinib	Docetaxel	489	NR	21	1.12 (0.89–1.40)	1.10 (0.83–1.43)	1.23 (0.81–1.84)	0.93 (0.58–1.48)	1.13 (0.87–1.45)
Cappuzzo et al. (19)	Unknown	SATURN	>1	Erlotinib	Placebo	889	60/60	11.4/11.5	0.81 (0.70–0.95)	0.88 (0.74–1.05)	0.64 (0.46–0.91)	0.69 (0.45–1.05)	0.84 (0.71–0.99)
Fukuoka et al. (20)	Unknown	IPASS	1	Gefitinib	Carboplatin plus paclitaxel	1,217	57/57	17	0.90 (0.79–1.02)	0.77 (0.59–1.02)	0.93 (0.81–1.08)	0.90 (0.78–1.03)	0.99 (0.62–1.60)
Lee et al. (21)	Unknown	TOPICAL	1	Erlotinib	Placebo	670	77/77	NR	0.94 (0.81–1.10)	Female vs. male 0.81 (0.59–1.01)		Never vs. ever 0.64 (0.36–1.14)	
Pérol et al. (22)	Unknown	IFCT-GFPC 0502	>1	Erlotinib	Observation	310	56.4/59.8	25.6	0.87 (0.68–1.13)	0.88 (0.66–1.18)	0.89 (0.52–1.53)	0.83 (0.34–2.01)	0.88 (0.68–1.15)
Kelly et al. (23)	Wild-type	PDX-012	>1	Erlotinib	Pralatrexate	201	62/63	NR	1.19 (0.88–1.64)	1.08 (0.75–1.56)	1.64 (0.92–2.94)	NR	NR
Miller et al. (24)	Unknown	LUX-Lung 1	>1	Afatinib	Placebo	585	58/59	NR	1.08 (0.86–1.35)	1.17 (0.83–1.65)	1.02 (0.76–1.37)	1.20 (0.90–1.61)	0.43 (0.31–0.61)
Ciuleanu et al. (25)	Unknown	TITAN	>1	Erlotinib	Docetaxel or pemetrexed	424	59/59	27.9/24.8	0.96 (0.78–1.19)	0.86 (0.67–1.10)	1.23 (0.78–1.94)	0.86 (0.49–1.51)	0.93 (0.78–1.11)
Garassino et al. (26)	Wild-type	TAILOR	>1	Erlotinib	Docetaxel	219	66/67	33	1.27 (0.95–1.69)	1.18 (0.84–1.67)	1.47 (0.84–2.56)	1.69 (0.89–3.23)	1.12 (0.81–1.54)
Inoue et al. (27)	Mutation	NEJ002	1	Gefitinib	Carboplatin plus paclitaxel	228	NR	23	0.89 (0.63–1.24)	0.92 (0.53–1.61)	0.88 (0.57–1.35)	0.88 (0.57–1.37)	0.98 (0.58–1.65)
Ellis et al. (28)	Unknown	NCIC CTG BR.26	>1	Dacomitinib	Placebo	720	63.5/65.5	23.4/24.4	1.00 (0.83–1.21)	NR	NR	0.74 (0.56–0.98)	1.13 (0.91–1.40)
Gregorc et al. (29)	Unknown	PROSE	>1	Erlotinib	Pemetrexed or docetaxel	263	66/64	32.4	1.15 (0.83–1.59)	Female vs. male 0.90 (0.64–1.27)		Never vs. ever 0.80 (0.51–1.27)	
Li et al. (30)	Wild-type	NR	>1	Erlotinib	Pemetrexed	123	54.3/55.1	14.7	1.01 (0.66–1.54)	1.24 (0.73–2.11)	0.64 (0.31–1.33)	0.81 (0.34–1.90)	1.10 (0.68–1.80)
Karachaliou et al. (31)	Unknown	EURTAC	1	Erlotinib	Cisplatin plus docetaxel or gemcitabine	97	NR	49.4	0.71 (0.45–1.12)	Female vs. male 0.96 (0.59–1.56)		NR	NR
Zhou et al. (32)	Mutation	OPTIMAL	1	Erlotinib	Gemcitabine plus carboplatin	154	57/59	25.9	1.19 (0.83–1.71)	1.31 (0.75–2.31)	1.20 (0.74–1.93)	1.44 (0.93–2.24)	0.85 (0.44–1.64)
Wu et al. (33)	Mutation	ENSURE	1	Erlotinib	Gemcitabine plus cisplatin	217	57.5/56	28.9/27.1	0.91 (0.63–1.31)	0.86 (0.45–1.64)	0.92 (0.59–1.44)	0.99 (0.65–1.52)	0.68 (0.32–1.43)
Yang et al. (34)	Mutation	LUX-Lung 3 and LUX-Lung 6	1	Afatinib	Pemetrexed-cisplatin or gemcitabine-cisplatin	631	60/59	41/33	0.81 (0.66–0.99)	0.71 (0.51–0.99)	0.84 (0.65–1.09)	0.72 (0.57–0.92)	1.02 (0.69–1.50)
Zhao et al. (35)	Unknown	INFORM	>1	Gefitinib	Placebo	296	55/55	17.8	0.88 (0.68–1.14)	0.89 (0.64–1.24)	0.93 (0.61–1.41)	0.94 (0.65–1.35)	0.82 (0.57–1.19)
Shi et al. (36)	Mutation	CONVINCE	1	Icotinib	Cisplatin plus pemetrexed	285	56	39.6	1.03 (0.76–1.39)	1.19 (0.69–2.04)	0.96 (0.67–1.39)	1.02 (0.72–1.45)	1.20 (0.64–2.27)

*EGFR, epidermal growth factor receptor; TKI, tyrosine kinase inhibitor; HR, hazard ratio; CI, confidence interval; NR, none reported*.

In particular, Ellis et al. (28) did not report the HR for sex, and Kelly et al. (23) and Karachaliou et al. (31) did not report the HRs for smoking status that we needed. Lee et al. (21) Gregorc et al. (29) and Karachaliou et al. (31) reported the interaction HR of sex (female vs. male) in their subgroup analysis. Additionally, Lee et al. (21) and Gregorc et al. (29) reported the interaction HR of smoking status (never vs. ever smokers) in their subgroup analysis. For these interaction HRs with 95% CIs, we extracted and applied them in our meta-analysis directly (Table 1).

### Primary Analysis

**Figure 2:**
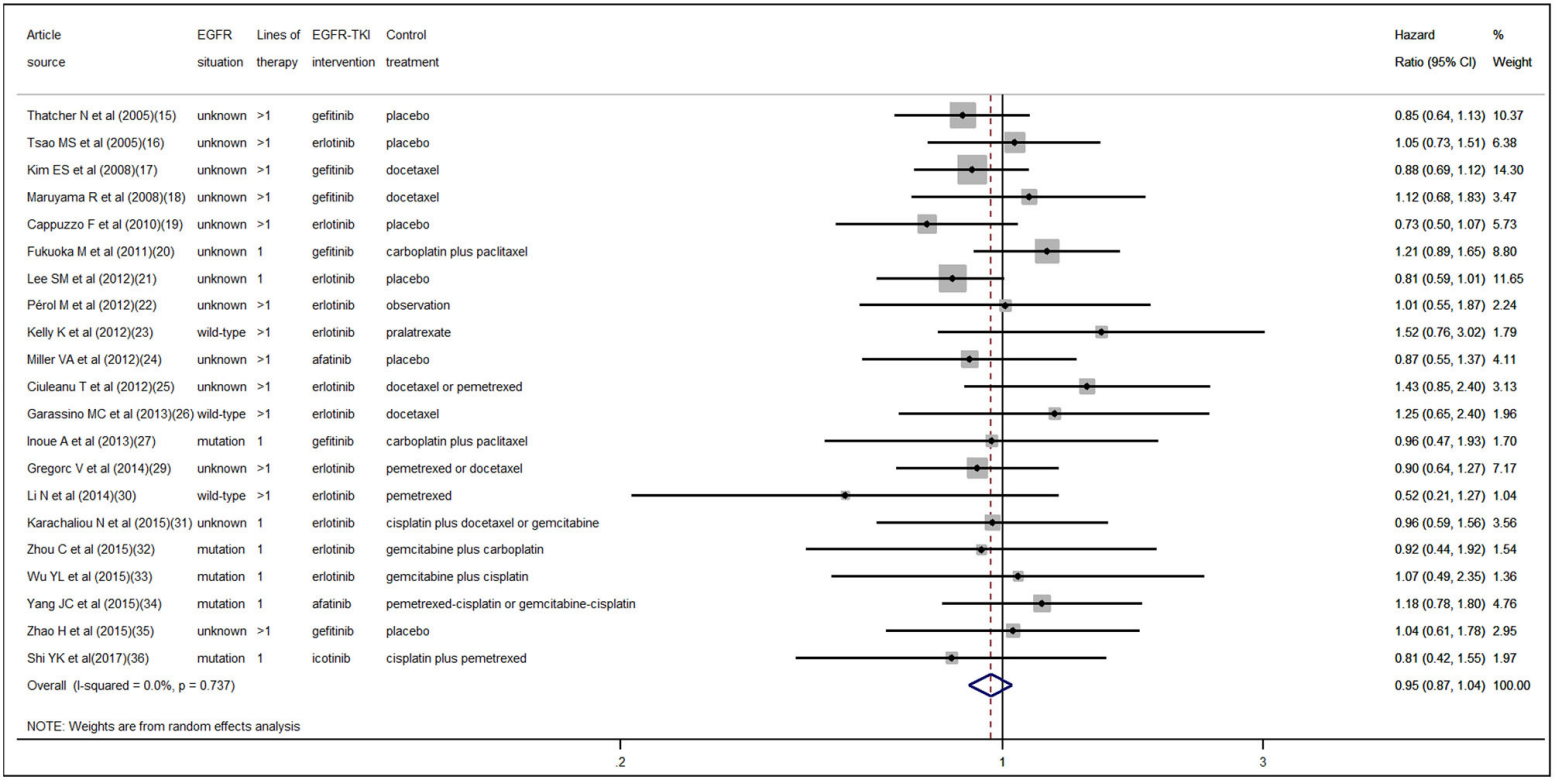
Forest plot of the pooled analysis of the interaction hazard ratios of sex.

**Figure 3:**
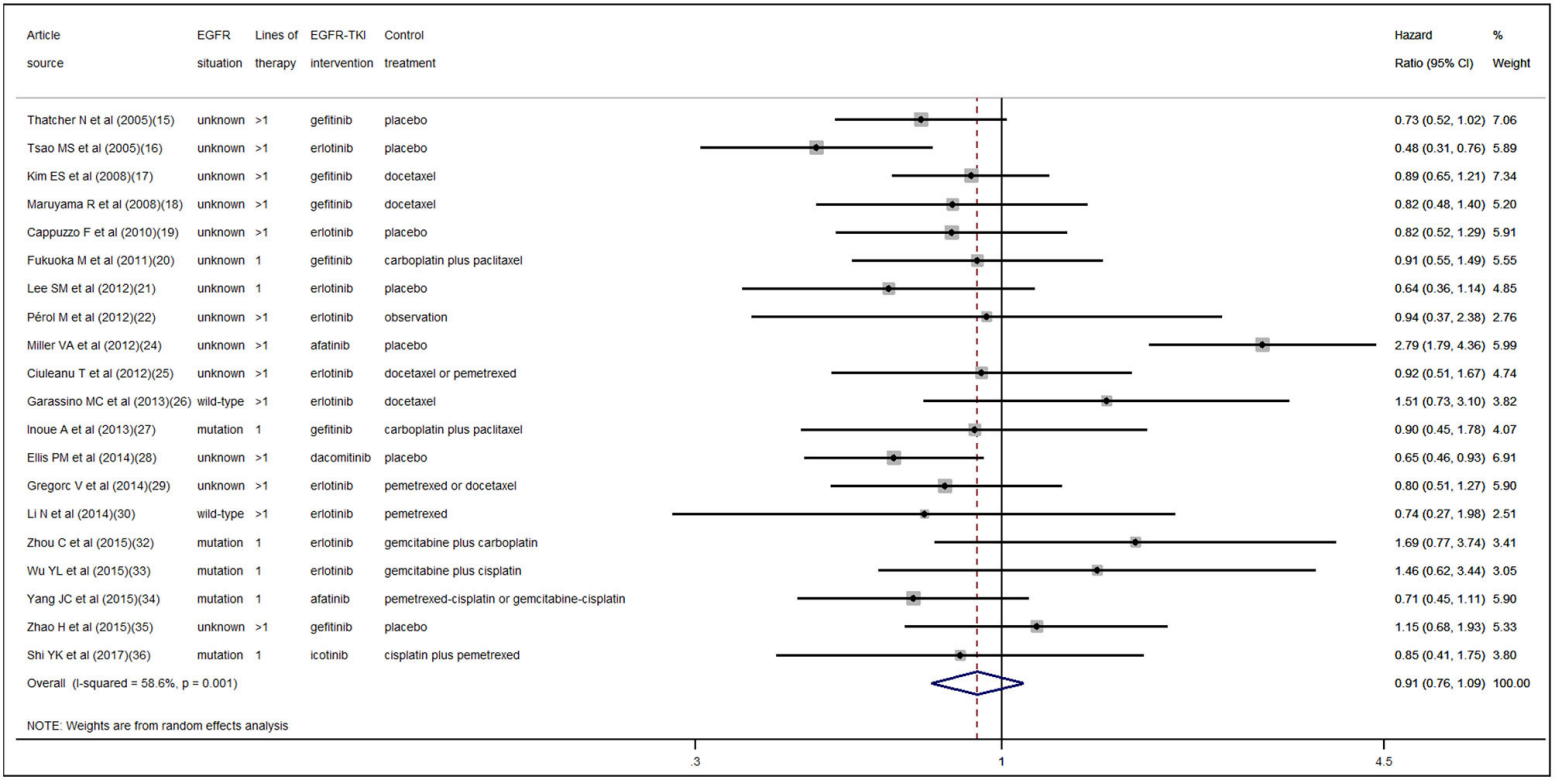
Forest plot of the pooled analysis of the interaction hazard ratios of smoking status.

According to the pooled result of overall HRs, we found that the therapeutic effect of EGFR-TKI intervention was better than that of the control treatment (pooled HR 0.94, 95% CI 0.89–1.00, *P* = 0.05) in NSCLC (Figure S1 in the Supplementary Material). When pooling the interaction HRs of sex, the results showed no statistically significant efficacy difference in EGFR-TKI intervention between females and males (pooled HR 0.95, 95% CI 0.87–1.04, *P* = 0.30) (Figure 2). Similarly, based on the pooled interaction HR of smoking status, there was also no statistically significant efficacy difference in EGFR-TKI intervention between never smokers and ever smokers (pooled HR 0.91, 95% CI 0.76–1.09, *P* = 0.31) (Figure 3).

### Heterogeneity and Publication Bias

Statistically significant interstudy heterogeneity was identified among both overall HRs (I-squared = 38.4%, *P* = 0.04) and smoking status interaction HRs (I-squared = 58.6%, *P* < 0.01) but not in sex interaction HRs (I-squared = 0.00%, *P* = 0.74) (Figure S1 in the Supplementary Material, Figures 2, 3). Both Egger and Begg tests indicated no evidence of publication bias.

### Subgroup Analysis

We further performed subgroup analyses according to EGFR status (unknown, wild-type, and mutation), lines of therapy (>1 and 1), EGFR-TKI intervention (gefitinib, erlotinib, and others), and control treatment (placebo, chemotherapy, and others). According to the pooled interaction HRs of sex, we found a statistically significant OS advantage for females compared with males only for the EGFR-TKI intervention compared with the control placebo treatment (pooled HR 0.86, 95% CI 0.75–1.00, *P* = 0.04), while the other groups showed no statistically significant difference (Table 2). Furthermore, as shown in Table 3, no statistically significant difference was determined in any of the subgroups according to the pooled interaction HRs of smoking status.

**Table 2:** Differences in efficacy of EGFR-TKI therapy in females and males by subgroup.

**Subgroups**		**Number of trials**	**Pooled interaction HR**	**P-interaction**
Overall		21	0.95 (0.87–1.04)	0.30
EGFR situation	Unknown	13	0.94 (0.85–1.03)	0.19
	Wild-type	3	1.06 (0.60–1.89)	0.83
	Mutation	5	1.02 (0.78–1.34)	0.86
Lines of therapy	>1	13	0.94 (0.84–1.05)	0.26
	1	8	0.98 (0.84–1.15)	0.82
EGFR-TKI intervention	Gefitinib	6	0.97 (0.84–1.11)	0.64
	Erlotinib	12	0.93 (0.82–1.07)	0.31
	Others	3	0.98 (0.74–1.30)	0.91
Control treatment	Placebo	6	0.86 (0.75–1.00)	0.04
	Chemotherapy	13	1.01 (0.89–1.14)	0.89
	Others	2	1.21 (0.77–1.91)	0.42

**Table 3:** Differences in efficacy of EGFR-TKI therapy according to smoking status by subgroup.

**Subgroups**		**Number of trials**	**Pooled interaction HR**	**P-interaction**
Overall		20	0.91 (0.76–1.09)	0.31
EGFR situation	Unknown	13	0.87 (0.69–1.10)	0.24
	Wild-type	2	1.15 (0.58–2.27)	0.68
	Mutation	5	0.95 (0.69–1.32)	0.77
Lines of therapy	>1	13	0.91 (0.71–1.17)	0.46
	1	7	0.87 (0.69–1.10)	0.25
EGFR-TKI intervention	Gefitinib	6	0.86 (0.73–1.03)	0.10
	Erlotinib	10	0.86 (0.67–1.10)	0.23
	Others	4	1.03 (0.50–2.11)	0.95
Control treatment	Placebo	8	0.88 (0.59–1.29)	0.50
	Chemotherapy	12	0.91 (0.77–1.06)	0.22

## Discussion

OS and PFS are the main endpoints in clinical trials of cancer. It is well-known that PFS is not in line with OS in many cases, (37) and in such cases, cancer patients may not obtain benefit from OS even though they have an improved PFS. To reduce time, save costs, and improve drug development efficiency, an increasing number of cancer clinical trials have set the research endpoint to PFS. However, compared with PFS, OS is simple, reliable, straightforward, clear, and accurate in the evaluation of the endpoint of cancer patients. Therefore, more attention should be paid to OS. In our current study, we studied the impact of sex and smoking status on the efficacy of EGFR-TKI therapy in terms of OS in NSCLC patients and obtained meaningful findings.

We first demonstrated the advantage in OS for NSCLC patients who received EGFR-TKI intervention compared with other systemic therapies. Thereafter, we found no significant OS differences for EGFR-TKI intervention between the sexes and smoking status compared with other systemic therapies. Finally, in the subgroup analyses, when compared with placebo, we demonstrated that female NSCLC patients who received EGFR-TKI therapy had a longer OS than males. However, smoking status showed no significant effect on the efficacy of EGFR-TKI treatment in terms of the OS of NSCLC patients in all of our subgroup analyses.

In recent years, significant sex-based differences in biology, epidemiology and treatment responses have become evident (38). There are sex-related differences in the clinicopathological characteristics of NSCLC patients, and female sex is a separate advantage survival prognostic factor (4). Consistently, after adjustments for other prognostic factors, males with NSCLC have a poorer prognosis than females (39). As NSCLC is considered a sex-related disease, further investigation is warranted to advance the treatment of NSCLC patients.

In a previous meta-analysis conducted by Lee et al. (40), EGFR-TKI treatment significantly prolonged PFS for female compared with male NSCLC patients with EGFR mutations. Afterwards, another meta-analysis also performed by Lee et al. (41) further concluded that, there was no difference in OS between EGFR-TKI and chemotherapy, as well as no difference in OS between female and male EGFR mutation-positive NSCLC patients. However, in our current study, we found that NSCLC patients who received EGFR-TKI intervention had longer OS than those who received other systemic therapies, although no significant OS differences for EGFR-TKI intervention were found between the sexes. In the subgroup analysis of the placebo control group, we demonstrated that female NSCLC patients who received EGFR-TKI therapy had a longer OS than males.

For the studies on chemotherapy in patients with NSCLC, Wakelee et al. (42) reported that women had a 1.9-month statistically significant improvement in OS compared with men. Wheatley-Price et al. (43) also concluded that females had a higher response rate to chemotherapy and a longer OS than males. For our current study, when the control group was treated with chemotherapy, it significantly biased our judgment of the difference in efficacy of EGFR-TKI between the sexes. When we removed the interference of chemotherapy and other factors in the subgroup analysis and compared EGFR-TKIs with the standard placebo, we found that the efficacy of EGFR-TKIs in female patients was significantly better than that in male patients. These results indicate that there is indeed a sex difference in the efficacy of EGFR-TKIs in patients with NSCLC.

It is well-known that tobacco smoking is an important cause of the development and progression of NSCLC. The incidence of EGFR mutations in NSCLC differs according to smoking history (44). EGFR mutations are highly prevalent in never smokers with NSCLC (45). Current smoking is an independent poor prognostic factor for survival for advanced non-squamous NSCLC patients without EGFR mutations who undergo pemetrexed continuation maintenance therapy (46). In addition, according to a recently reported large population-based study, NSCLC in never smokers was found to be clinically different from smoking-associated NSCLC, and the study also concluded that the OS in never-smokers was longer than that in smokers (47).

The impact of smoking status on the efficacy of EGFR-TKIs in terms of PFS in NSCLC is contradictory according to previous meta-analyses (9, 10, 40). For the meta-analyses that studied OS, Sohn et al. (48) reported that, compared with chemotherapy or placebo, receiving EGFR-TKI therapy appeared to show longer OS among patients with NSCLC for never smokers than that seen in ever smokers. In contrast, Lee et al. (41) found no difference in OS according to smoking status for NSCLC patients who underwent EGFR-TKI treatment compared with chemotherapy. We consider this contradictory phenomenon to be due to the different inclusion criteria and the different number of included studies. However, in our current study, we found no significant OS differences for EGFR-TKI intervention compared with other systemic therapies between different smoking statuses in NSCLC patients, and further subgroup analyses also showed that smoking status had no significant effect on the efficacy of EGFR-TKI treatment.

In summary, since sex and smoking status are the two main clinical features of lung cancer, our current research has important guiding significance for the clinical treatment of lung cancer. Our results suggest that we do not need to worry that smoking status will affect the efficacy of EGFR-TKIs and that EGFR-TKIs will have better efficacy in female patients than in male patients. However, on the other hand, the efficacy of EGFR-TKIs in male patients is not so ideal, indicating that more treatment options for male lung cancer patients need to be further developed in the future.

Our current study has several limitations. First, as a meta-analysis, it relies on published results rather than the individual data of patients. Second, those excluded studies that lack published sex and smoking status subgroup analysis data may also contain potential differences. Finally, aside from sex and smoking status, differences in OS outcomes may be influenced by other non-pharmacological factors.

## Conclusions

Two main conclusions can be drawn from our current meta-analysis. The first is that the efficacy of EGFR-TKI therapy for NSCLC patients is sex-dependent, and females have a longer OS advantage than males. The second point is that smoking status has no effect on the efficacy of EGFR-TKI therapy in terms of the OS of NSCLC patients.

## Data Availability Statement

All datasets generated for this study are included in the article/Supplementary Material.

## Author Contributions

JX and QC: concept and design. JX and LZ: acquisition, analysis or interpretation of data, and statistical analysis. JX and BH: drafting of the manuscript. JX, BH, and QC: critical revision of the manuscript for important intellectual content. BH and QC: administrative, technical or material support, and study supervision. All authors contributed to the article and approved the submitted version.

## Conflict of Interest

The authors declare that the research was conducted in the absence of any commercial or financial relationships that could be construed as a potential conflict of interest.

## Abbreviations

EGFR: epidermal growth factor receptor
EGFR-TKIs: epidermal growth factor receptor-tyrosine kinase inhibitors
NSCLC: non-small-cell lung cancer
OS: overall survival
HR: hazard ratio
CI: confidence interval
PFS: progression-free survival.
