# Supplementary material for: Impact of Sex and Smoking on the Efficacy of EGFR-TKIs in Terms of Overall Survival in Non-small-Cell Lung Cancer: A Meta-Analysis
Source: Front Oncol. 2020 Aug 25;10:1531. doi: 10.3389/fonc.2020.01531 (PMC7477328; doi:10.3389/fonc.2020.01531)

**Supplementary Contents**

**Impact of sex and smoking on the efficacy of EGFR-TKIs in terms of overall survival in** **non-small-cell lung cancer: a** **meta-analysis**

**Supplementary content 1**

**Search terms used in databases**

**Search terms used in the database of PubMed:**

((lung[title/abstract] AND cancer[title/abstract]) OR "lung neoplasms"[MeSH Terms] OR "lung neoplasms"[MeSH Terms]) AND (survival[title/abstract] OR survivals[title/abstract] OR outcome[title/abstract] OR outcomes[title/abstract] OR "survival"[MeSH Terms]) AND (HR[title/abstract] OR HRs[title/abstract] OR (hazard[title/abstract] AND (ratio[title/abstract] OR ratios[title/abstract]))) AND (EGFR[title/abstract] OR "epidermal growth factor receptor"[title/abstract] OR "erbb receptors"[MeSH Terms] OR "erbb receptors"[MeSH Terms] OR "erbb receptors"[MeSH Terms]) AND (((randomized[title/abstract] OR randomised[title/abstract] OR random[title/abstract] OR controlled[title/abstract]) AND (trial[title/abstract] OR trials[title/abstract] OR test[title/abstract] OR tests[title/abstract])) OR "randomized controlled trials as topic"[MeSH Terms] OR "clinical trials as topic"[MeSH Terms]) NOT (meta[title] OR meta-analysis[title] OR review[title] OR "meta-analysis as topic"[MeSH Terms]) AND ((Clinical Study[ptyp] OR Clinical Trial[ptyp] OR Multicenter Study[ptyp]) AND English[lang])

**Search terms used in the database of Cochrane Library:**

#1：MeSH descriptor: [Lung Neoplasms] explode all trees

#2：MeSH descriptor: [ErbB Receptors] explode all trees

#3：(survival OR survivals OR outcome OR outcomes):ti,ab,kw

#4：(HR OR HRs OR (hazard ratio) OR (hazard ratios)):ti,ab,kw

#5：(meta OR meta-analysis OR review):ti

#6：(#1 AND #2 AND #3 AND #4) NOT #5

**Search terms used in the database of Embase:**

('lung cancer'/exp OR 'lung cancer') AND ('epidermal growth factor receptor'/exp OR 'epidermal growth factor receptor') AND ('tyrosine kinase inhibitor'/exp OR 'tyrosine kinase inhibitor') AND ('survival'/exp OR survival) AND ('clinical study'/exp OR 'clinical study') AND ('hazard'/exp OR hazard OR HR:ab,ti OR HRs:ab,ti) NOT (meta:ti OR 'meta analysis':ti OR review:ti) AND ([controlled clinical trial]/lim OR [randomized controlled trial]/lim) AND ([article]/lim OR [article in press]/lim) AND [english]/lim AND [humans]/lim AND [clinical study]/lim

**Search terms used in the database of Scopus:**

TITLE-ABS-KEY(lung) AND TITLE-ABS-KEY(cancer) AND TITLE-ABS-KEY(survival OR survivals OR outcome OR outcomes) AND TITLE-ABS-KEY(EGFR OR "epidermal growth factor receptor") AND TITLE-ABS-KEY("tyrosine kinase inhibitor" OR TKI OR TKIs) AND TITLE-ABS-KEY(HR OR HRs OR "hazard ratio" OR "hazard ratios") AND TITLE-ABS-KEY(randomized OR randomised OR random OR controlled) AND TITLE-ABS-KEY(trial OR trials OR test OR tests) AND NOT TITLE(meta OR meta-analysis OR review) AND (LIMIT-TO (DOCTYPE, "ar")) AND (LIMIT-TO(SUBJAREA, "MEDI") OR LIMIT-TO(SUBJAREA, "PHAR") OR LIMIT-TO(SUBJAREA, "MULT")) AND (LIMIT-TO(EXACTKEYWORD, "Human")) AND (LIMIT-TO(LANGUAGE, "English")) AND (LIMIT-TO(SRCTYPE, "j"))

**Supplementary content 2**

**Table S1.** The Jadad quality scores of included studies

| Article source | Year of publication | Randomization | Blinding | Withdrawals and dropouts | Total score |
| --- | --- | --- | --- | --- | --- |
| Thatcher N et al (15) | 2005 | 2 | 2 | 1 | 5 |
| Tsao MS et al (16) | 2005 | 2 | 2 | 1 | 5 |
| Kim ES et al (17) | 2008 | 2 | 0 | 1 | 3 |
| Maruyama R et al (18) | 2008 | 2 | 0 | 1 | 3 |
| Cappuzzo F et al (19) | 2010 | 2 | 2 | 1 | 5 |
| Fukuoka M et al (20) | 2011 | 2 | 0 | 1 | 3 |
| Lee SM et al (21) | 2012 | 2 | 2 | 1 | 5 |
| Pérol M et al (22) | 2012 | 2 | 0 | 1 | 3 |
| Kelly K et al (23) | 2012 | 2 | 0 | 1 | 3 |
| Miller VA et al (24) | 2012 | 2 | 2 | 1 | 5 |
| Ciuleanu T et al (25) | 2012 | 2 | 0 | 1 | 3 |
| Garassino MC et al (26) | 2013 | 2 | 0 | 1 | 3 |
| Inoue A et al (27) | 2013 | 2 | 0 | 1 | 3 |
| Ellis PM et al (28) | 2014 | 2 | 2 | 1 | 5 |
| Gregorc V et al (29) | 2014 | 2 | 0 | 1 | 3 |
| Li N et al (30) | 2014 | 2 | 0 | 1 | 3 |
| Karachaliou N et al (31) | 2015 | 2 | 0 | 1 | 3 |
| Zhou C et al (32) | 2015 | 2 | 0 | 1 | 3 |
| Wu YL et al (33) | 2015 | 2 | 0 | 1 | 3 |
| Yang JC et al (34) | 2015 | 2 | 0 | 1 | 3 |
| Zhao H et al (35) | 2015 | 2 | 2 | 1 | 5 |
| Shi YK et al (36) | 2017 | 2 | 0 | 1 | 3 |

**Supplementary content 3**

**Figure S1.** Hazard ratios of death for NSCLC patients that conducted EGFR-TKI therapy compared with control treatment


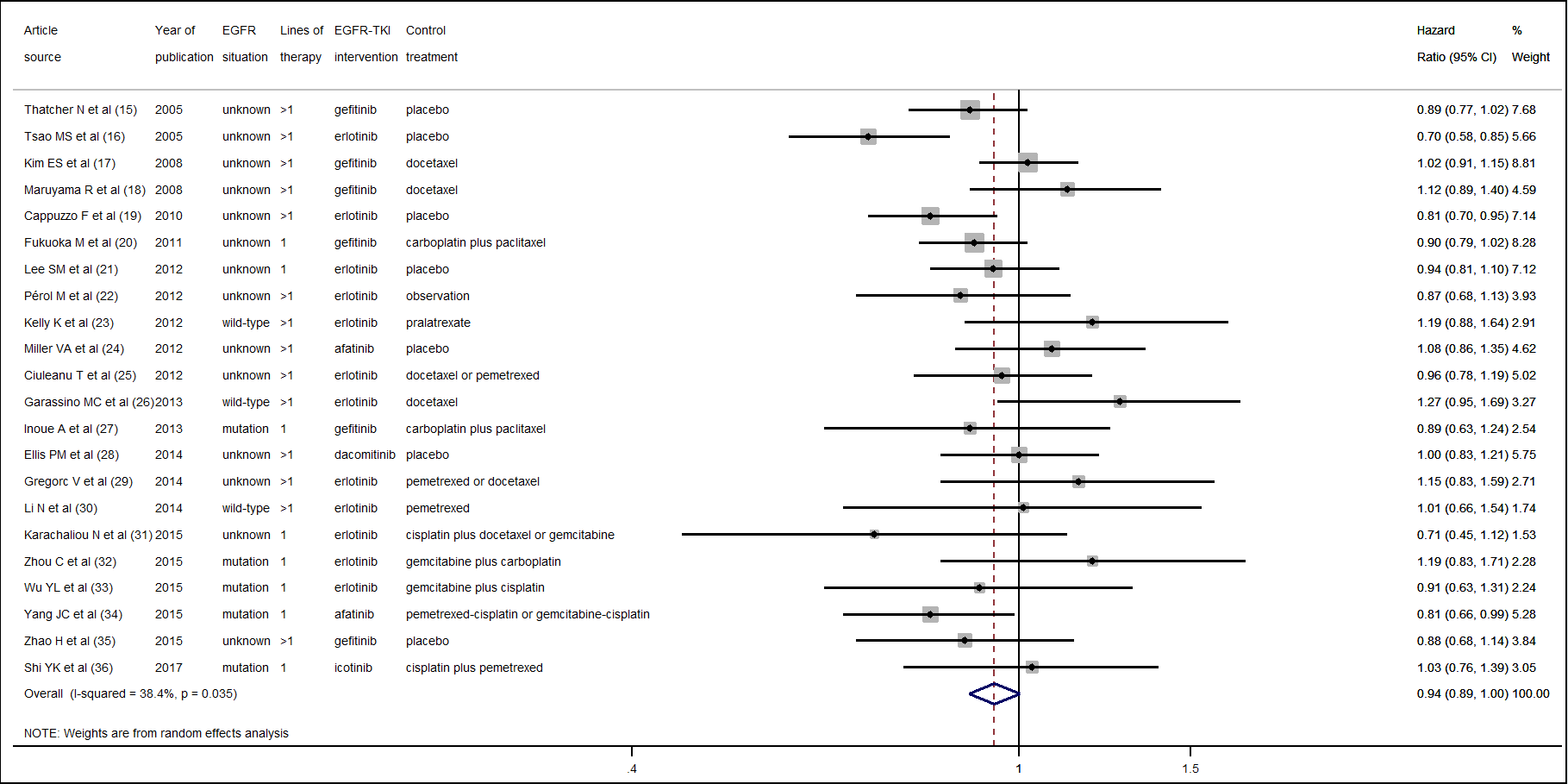

Supplement: Supplementary file 1 [file Data_Sheet_1.DOCX]
